# A systematic review and narrative synthesis of the psychometric properties and biopsychosocial correlates of the English version of the Intuitive Eating Scale-2

**DOI:** 10.1371/journal.pone.0349590

**Published:** 2026-05-21

**Authors:** Rebecca J. Linnett, Noelle Robertson, Stephanie J. Hubbard

**Affiliations:** 1 Department of Population Health Sciences, University of Leicester, Leicester, United Kingdom; 2 Department of Psychology and Vision Sciences, University of Leicester, Leicester, United Kingdom; The Chinese University of Hong Kong, HONG KONG

## Abstract

Intuitive eating is an adaptive eating approach characterised by having unconditional permission to eat when you are hungry, eating for physical rather than emotional reasons, relying on internal hunger and satiety cues, and honouring your health and practising gentle nutrition. The Intuitive Eating Scale-2 (IES-2) is currently the most commonly-used intuitive eating measure but, to date, there has not been a systematic review of how it performs psychometrically outside of the student sample in which it was developed. This systematic review aimed to assess the psychometric properties of the IES-2, including its associations with other variables, across all studies that used it as a measure of intuitive eating and reported psychometric data. MEDLINE, PsycINFO, Scopus and Web of Science were searched in April 2022, May 2024 and June 2025, identifying 90 papers from diverse populations including college students, people from the general population/community, and people seeking treatment for eating disorders or weight management. Results were presented and synthesised narratively, and risk of bias was assessed using two domains from the COSMIN Risk of Bias checklist. Findings suggest that the IES-2 has good construct validity but underperforms psychometrically in other areas such as response distribution, subscale inter-correlations and dimensionality, particularly in relation to the ‘Unconditional permission to eat’ subscale. Alternative factor structures were reported on and a three-factor solution excluding the ‘Unconditional permission to eat’ subscale was found to have promising results. The review contributes a comprehensive account of the biopsychosocial correlates of the IES-2, as well as identifying that studies relating to weight or eating disorders often report mean scores that tend towards the extremes of the scale. Limitations include the exclusion of non-English translations of the IES-2, and future reviews would benefit from being conducted in other languages. PROSPERO registration: CRD42022299436. Funder: ESRC (ES/P000711/1).

## 1. Background and rationale

Intuitive eating, also known as ‘normal eating’ or ‘non-dieting’ [1] is an eating approach that is characterised by four central features: 1) Having unconditional permission to eat when you are hungry, without considering certain foods ‘forbidden’; 2) Eating for physical rather than emotional reasons; 3) Relying on internal hunger and satiety cues to determine what, when, and how much to eat; and 4) ‘Body-food choice congruence’, which focuses on honouring your health and practising gentle nutrition – e.g., by choosing foods that promote energy and stamina, and that make you feel well [2]. Intuitive eating is not just the absence of pathological eating, but an adaptive eating approach that promotes health by developing an awareness of – and respect for – innate physiological cues [3]. The concept of intuitive eating was developed in 1995 by two registered dieticians in the United States, Evelyn Tribole and Elyse Resch, who developed the approach as a result of their experience working with clients who struggled with weight cycling and chronic dieting [4].

In terms of health benefits, there is evidence to suggest that intuitive eating is associated with lower levels of binge eating, disinhibited eating, obsessive-compulsive eating and disordered eating generally [5,6], lower cholesterol, body mass index (BMI) and blood pressure [1,7] and better glycaemic control amongst people with diabetes [8–11]. The psychological benefits of an intuitive eating approach are also clear; intuitive eating is associated with increased life satisfaction [5,12–14], self-esteem [12,13], positive body image, body appreciation and body esteem [5,7,12–15] and negatively associated with disordered eating and extreme or unhealthy weight loss practices [3,12].

The Intuitive Eating Scale-2 [IES-2] is a 23-item self-report questionnaire that was developed by a counselling psychologist, Tracy Tylka, based on the work of Tribole and Resch. The scale taps into all four of the dimensions of intuitive eating detailed above. Each item is scored on a five-point Likert scale (from ‘Strongly disagree’ to ‘Strongly agree’) and higher scores reflect higher levels of the construct. Although a third iteration of the scale has recently been developed [16], the IES-2 is still the most commonly-used measure of intuitive eating at present, superseding previous iterations of the scale [see 13, 17]. The IES-2 was developed with a sample of 1,405 women and 1,195 men across three studies [2]. The sample was made up entirely of college students from a regional campus of a large Midwestern university in the USA, was predominantly white (more than 75% in each sample) and ranged in age from [18–56].

In the 11 years since the publication of the IES-2, evidence relating to its psychometric properties and how it is associated with other variables of interest has accumulated from a large number of studies across many different sample types, including people with eating disorders [e.g., 18, 19], people with kidney disease [20], trans and nonbinary people [21], people with severe mental illness [22], older adults [23] and adolescents [24], as well as the usual general population and student samples. However, although there have been systematic reviews of the psychosocial correlates of intuitive eating as a construct [3,12], to date an exhaustive review of the IES-2’s psychometric properties has not been conducted and there is therefore no comprehensive picture of how it functions in these different samples or how it is associated with other indicators of psychological and physical health. This review aims to fill this gap by providing a comprehensive exploration of the different samples that the IES-2 has been used in and how its psychometric properties vary across different populations.

## 2. Research questions and aims

Given this gap in the literature, the aim of this review was to assess the psychometric properties of the IES-2 (including its associations with other variables) across all studies that used it as a measure of intuitive eating and reported at least one of the measurement properties of interest (as detailed in Section 3.1). The reason for doing this was to make it possible to evaluate the evidence currently available about the measure and assess its applicability across different sample types, with the aim of supporting decision-making around its future use.

To achieve this, this systematic review aimed to answer the following questions:

1. What are the psychometric properties of the Intuitive Eating Scale-2 across diverse populations and contexts?
2. How does intuitive eating, as measured by the Intuitive Eating Scale-2, relate to other variables associated with physical and psychological health?

## 3. Method

This review was guided by the COSMIN methodology for systematic reviews of outcome measurement instruments [25,26]. However, it should be noted that only some aspects of the COSMIN methodology were incorporated in this review as this methodology is developed for reviews of studies that have directly assessed the measurement properties of an instrument. As this review included any studies that had used the IES-2 as an outcome measure, certain elements of the COSMIN methodology were not relevant to this review or could not be adequately assessed (including content validity and some aspects of measurement invariance).

The protocol for this review was registered with PROSPERO on 06/01/2022 (CRD42022299436). Searches were initially run on 12/04/2022 and were run again between 13/05/2024 and 14/05/2024 and for a third time on 03/06/2025 to update the review prior to publication. This study is reported in compliance with PRISMA 2020 guidelines and a PRISMA 2020 checklist is provided in the S1 Table.

### 3.1. Eligibility criteria

In order to be included in this review, the study must:

1 Have used the full 23-item Intuitive Eating Scale-2 in English [2]. Studies that used non-English translations, other versions of the Intuitive Eating Scale [13,17] or that only used certain subscales were not included.
2 Have been available in full-text
3 Have used the correct scoring procedure for the IES-2 items
4 Have provided information about at least one of the following psychometric properties:
  a Scale and/or subscale means
  b Subscale intercorrelations
  c Relationship to other variables associated with psychological or physical health (correlational and/or longitudinal)
  d Sex differences
  e Construct validity, consisting of:
    i Convergent validity – whether the measure correlates with other variables that theoretically should be related to it
    ii Discriminant validity – whether the measure is unrelated to variables that are not expected to be associated with it
    iii Known-groups validity – whether the measure is sensitive enough to detect known differences between certain groups
  f Reliability (e.g., test-retest, split half, internal consistency (α or Ω))
  g Factor analysis and/or factor structure

Systematic reviews, meta-analyses, literature reviews, unpublished theses and qualitative studies were not eligible to be included in the review, nor were any studies that did not present original empirical results. It is noted that the COSMIN guidelines [26] recommend that studies that only include the measure of interest as an outcome measurement should not be included in systematic reviews of patient-reported outcome measures as they only provide indirect evidence about measurement properties. However, in this instance preliminary searches suggested that there were insufficient studies that specifically focused on evaluation of the IES-2 and consequently it was necessary for the eligibility criteria to be widened to include all studies that had used the IES-2 and reported at least one psychometric property of interest.

Eligible studies published in languages other than English were not removed until full-text screening because Covidence records a reason for the exclusion of each paper at this stage which enabled us to keep a record of papers that were excluded for this reason. However, they were excluded at this stage and data were not extracted as there was not scope within the review’s time-frame or funding to allow for translation of non-English reports. Studies published in English but which used non-English translations of the IES-2 were also excluded from the review because of the potential for psychometric differences between the original and translated scales that were artefacts of the translation processes and quality rather than representing true cross-cultural variation [27,28].

### 3.2. Information sources

Searches for peer-reviewed journal articles were performed in Scopus, Web of Science, PsycINFO and MEDLINE. In addition, citation searches for the IES-2’s development paper [2] were performed in Scopus and Web of Science.

Due to the limited time-frame and funding of the review, it was decided that grey literature and non-indexed studies would not be included, and that authors would not be contacted about the possibility of unpublished studies or about any missing data in papers included in the review.

### 3.3. Search strategy

Initially, a citation search for the IES-2’s development paper [2] was performed. Papers were limited to those published from 2013 onward as this is when the IES-2 was published. While there were other scales of intuitive eating prior to this, the review is specifically focused on the IES-2 and therefore the searches were run from its year of publication. Keyword searches were structured as follows:

1. “intuitive eating” OR
2. “intuitive eating scale-2” OR
3. “intuitive eating scale 2” OR
4. “IES-2” OR
5. IES2

The search strategy was developed alongside a research librarian from the University of Leicester and was reviewed by the research team prior to implementation. The full search terms for each of the databases are detailed in Supplementary File 4.

### 3.4. Data management

Literature search results were uploaded to Covidence [29], an online systematic review program. Zotero reference management software was also used to manage references and to check for duplicates. In order to avoid “double counting”, author names, study dates, sample sizes and other characteristics and variables assessed were compared to ascertain whether multiple publications had arisen from the same study.

### 3.5. Selection process

Titles and abstracts yielded by the search were screened against the pre-defined eligibility criteria. Full reports were obtained for any titles that seemed to meet the criteria or any where the eligibility was uncertain. The full-text reports were then screened and a decision was made as to whether the inclusion criteria had been met. If studies were excluded, reasons for exclusion were recorded. If there was still uncertainty about study eligibility once the full-text report had been screened, study authors were contacted for clarification. All studies were screened by the first author (RL) and a subset of studies (10%) were reviewed independently by another member of the research team (the third author, SH) at each stage of the screening process. There was no disagreement between reviewers about the exclusion or inclusion of any of the studies, likely due to the tightly-defined eligibility criteria.

### 3.6. Data collection process

Standardised forms were created on Covidence and used to extract data from included studies. All data were extracted by the first author and any concerns discussed between all authors.

### 3.7. Data items

The following data items were extracted:

1 Publication details: Title, authors, publication date, journal published in, article metadata (volume, issue, pages, digital object identifier (DOI)), database(s) indexed in
2 Study characteristics: Type of study (e.g., intervention, cross-sectional), sample size, country/ies in which study took place, study date and duration, method of data collection (e.g., online, face-to-face, postal survey)
3 Participant characteristics: Sample type, gender distribution, age (mean, SD, range), ethnicity
4 Measurement properties (for the full scale and individual subscales):
  a Scale and/or subscale means
  b Subscale intercorrelations
  c Relationship to other variables (correlational and/or longitudinal) that pertain to physical or psychological health (these could not be established a priori as the aim of this element of the review was to explore the constructs that intuitive eating is associated with rather than assess its relationship to a limited number of pre-specified variables)
  d Sex differences
  e Construct validity (e.g., convergent, discriminant and/or known-groups validity)
  f Reliability
  g Factor analysis and/or factor structure

### 3.8. Data synthesis

Due to the exploratory nature of this review, the populations, outcomes and other characteristics of the studies included were deemed too heterogeneous for a meta-analysis to be feasible. Data were instead synthesised narratively as this approach can accommodate difference and allows for the descriptive exploration of heterogeneity [30].

### 3.9. Risk of bias

The risk of bias of each study was assessed using two domains from the COSMIN Risk of Bias checklist [25,26], specifically those relating to internal consistency and hypothesis testing for construct validity. The other COSMIN domains were not relevant to this review because the checklist is designed for studies that specifically evaluate the measurement properties of an instrument whereas the majority of the studies in this review only used the IES-2 as an outcome measure and did not report on its development. For each domain, studies were given a rating of either 1) Very good; 2) Adequate; 3) Doubtful; or 4) Inadequate quality. The study was then given an overall rating using the same scale, commensurate with the lowest domain rating it had received.

## 4. Results

### 4.1. Search results

**Fig 1:**
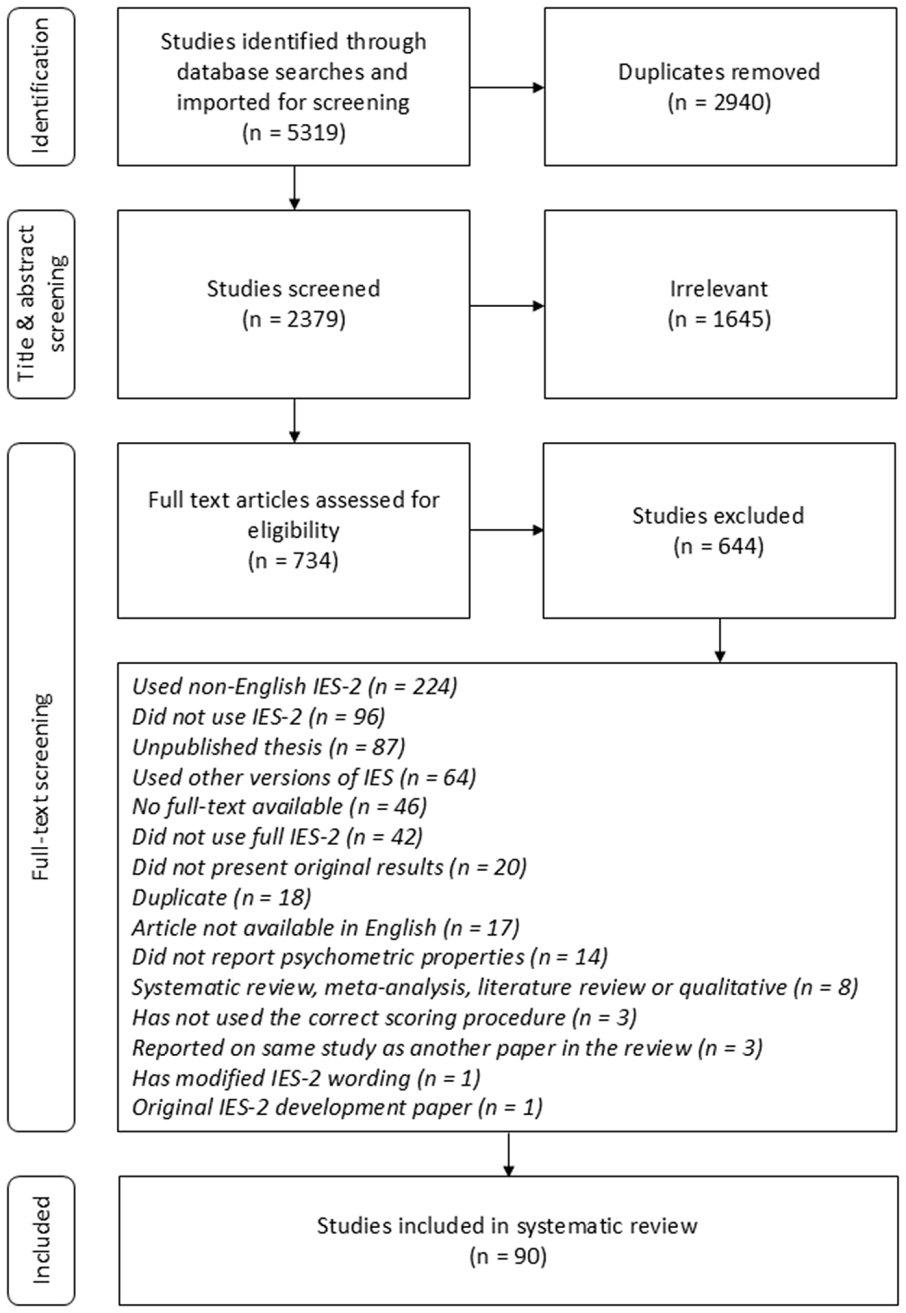
PRISMA diagram depicting study selection.

After removing duplicates, the search strategy yielded 2,379 articles published between the release of the IES-2 in 2013 and June 2025, when the final updated searches were conducted. Title and abstract screening led to the exclusion of 1,645 studies that were clearly irrelevant based on their titles or abstracts. An additional 644 studies were excluded after full-text review (see Fig 1 for a detailed overview of the study selection process). The most common reason for exclusion was the use of a non-English version of the IES-2 (224 studies). Other reasons included not using the IES-2 at all (96 studies), being an unpublished thesis (87 studies), or employing a different English-language measure of intuitive eating (64 studies), such as the original Intuitive Eating Scale [17] or the first version developed by Tracy Tylka [13]. The original IES-2 development paper was also excluded, as the review focused on studies that used the measure post-development. In total, 90 studies were included in the final review.

### 4.2. Quality assessment

Of the 90 studies included in the review, 66 (73.3%) were rated as ‘Very good’ and 24 (26.7%) were rated as ‘Inadequate’. The predominant reason for a study receiving an ‘Inadequate’ rating was for failing to calculate an internal consistency statistic for each subscale of the IES-2 (15 out of the 22 studies rated ‘Inadequate’ received this rating solely for this reason) – see Section 5.3 for further discussion.

### 4.3. Description of studies included

See Table 1 for a summary of the participant groups, countries of recruitment and largest ethnic groups of the reviewed studies and Table 2 for a summary of each paper included in the review. Mean participant ages ranged from a low of 15.14 years (*SD* = 1.43 years) [24], in one of only two studies to include adolescents, to a high of 68.8 years (*SD* = 6.3 years) [23] in a study focused on older adults. Nearly half of the studies had a mean age of 25 and under, likely due to the large proportion of university/college student samples. Twenty-four out of 90 studies (26.7%) recruited only women, two recruited only men and in all but seven of the remaining studies that reported the gender distribution of their participants, women made up more than 50% of the sample.

**Table 1:** Summary of study characteristics.

	n (%)
Participant group	
University/college students	29 (32.2)
General population/community	22 (24.4)
High(er) BMI/bariatric surgery	8 (8.9)
Eating disorder treatment	7 (7.8)
Other/unspecified	24 (26.7)
Country of recruitment	
USA	54 (60.0)
Australia and/or New Zealand	5 (5.6)
UK	5 (5.6)
Canada	4 (4.4)
Turkey	1 (1.1)
Malaysia	1 (1.1)
USA & Brazil^†^	1 (1.1)
Other/unspecified	19 (21.1)
Largest ethnic group	
White/Caucasian	60 (66.7)
Black/African American	5 (5.6)
Australian	2 (2.2)
Hispanic	2 (2.2)
Cuban	1 (1.1)
European	1 (1.1)
Chinese	1 (1.1)
Greek Cypriot	1 (1.1)
Asian American	1 (1.1)
New Zealand European	1 (1.1)
Other/not specified	15 (16.7)

† The data from the Brazilian arm was not included as it used a non-English version of the IES-2.

**Table 2:** Summary of published studies using the Intuitive Eating Scale-2 included in the review.

Study, country, largest ethnic group	Sample type	Age	Gender	N	Psychometric properties assessed	Biopsychosocial correlate(s)
Anastasiades & Argyrides [31] *Greek Cypriot*	Convenience sample (focus on orthorexia)	$\bar{x}$ = 40.24 yrs (range of 18–79 yrs)	62.0% female	834	Mean scale scores Internal consistency	Healthy orthorexia (+) Orthorexia nervosa (-) Body appreciation (+) Functionality appreciation (+) Embodiment (+) BMI (-)
Anastasiades & Argyrides [32]	General population sample (focus on orthorexia)	$\bar{x}$ = 40.31 yrs (range of 18–77 yrs)	61.7% female	814	Mean scale scores Internal consistency	–
Babbott et al. [33] *Australia & New Zealand* *Australian*	Seeking eating disorder treatment	$\bar{x}$ = 23.5 yrs	95.3% female	569	Mean scale scores Subscale inter-correlations Construct validity Internal consistency Dimensionality	Anxiety (-) Disordered eating (-) Depression (-) Stress (-)
Barad et al. [34] *USA*	Undergraduate students	$\bar{x}$ = 20 yrs (range of 19–21 yrs)	75.4% female	293	–	Fruit & veg intake (+/-)
Barney et al. [35] *USA* *White/Caucasian*	Women admitted to eating disorder treatment	$\bar{x}$ = 21.47 yrs	100% female	175	Mean scale scores Subscale inter-correlations Internal consistency	Anxiety (-) Negative body image (-) Depression (-) Disordered eating (-)
Bazo Perez et al. [36] *USA* *Hispanic*	Young adults	$\bar{x}$ = 21.99	90.2& female	620	Internal consistency Dimensionality	–
Belon et al. [37] *USA* *White/Caucasian*	College women	$\bar{x}$ = 20 yrs (range of 18–38 yrs)	100% female	352	Mean scale scores Internal consistency Dimensionality	–
Boucher et al. [38] *New Zealand* *NZ European*	Overweight women	$\bar{x}$ = 44.8 yrs	100% female	40	Mean scale scores Internal consistency	–
Braun et al. [39] *USA* *White/Caucasian*	Healthy people who identified as stressed, consumed <5 portions of fruit/veg a day	$\bar{x}$ = 39.4 yrs	70.5% female	78	Mean scale scores Internal consistency	–
Braun et al. [40] *USA* *White/Caucasian*	Adults who identified as stressed, consumed <5 portions of fruit/veg a day	$\bar{x}$ = 39.4 yrs	69.3% female	75	Mean scale scores Sex differences Internal consistency	BMI (-) Internalised weight stigma (-)
Braun et al. [41]	Women aged 18–65 years, BMI 40–55, elevated internalised weight stigma	$\bar{x}$ = 48.8 yrs	100% female	28	Mean scale scores	–
Brochu et al. [42] *White/Caucasian*	General population	$\bar{x}$ = 26.34 yrs (range of 18-80 yrs)	67% female	178	Mean scale scores Internal consistency	Body appreciation (+) Functionality appreciation (+) Weight bias internalisation (-)
Burnette & Mazzeo [43] *USA* *White/Caucasian*	Women college students with below clinical threshold ED behaviours	$\bar{x}$ = 20.11 yrs	100% female	71	Mean scale scores Internal consistency	–
Cole et al. [44] *USA*	Overweight military service members, family members or retirees	$\bar{x}$ = 50.2 yrs	71.4% female	56	Mean scale scores Internal consistency	–
Cook-Cottone et al. [45] *White/Caucasian*	Adult women in active recovery from an eating disorder	$\bar{x}$ = 36.69 yrs (range of 18-65 yrs)	100% female	277	Mean scale scores Internal consistency	–
Craven & Fekete [46] *USA* *White/Caucasian*	Female college students	$\bar{x}$ = 20.1 yrs	100% female	196	Mean scale scores Internal consistency	Binge eating (-) BMI (-) Weight-related shame & guilt (-)
Dakin et al. [47] *UK* *White/Caucasian*	General public and people involved in a weight management programme	$\bar{x}$ = 50.41 yrs	75.6% female	1660	Subscale inter-correlations Internal consistency	Eating self-regulation (+/-) Dietary restraint (+/-) External eating (+/-) Power of food (-) Positive emotional eating (+/-) Negative emotional eating (-) Emotional overeating (-) Satiety responsiveness (+) Emotional undereating (+) Food responsiveness (-) Binge eating (-) Disinhibition (-) Susceptibility to hunger (-)
Dakin et al. [48] *UK* *White*	General population (GP) and people undergoing weight management (WM)	GP: $\bar{x}$ = 45.9yrs WM: $\bar{x}$ = 55.6 yrs Range of 18–88 yrs	GP: 50.5% female WM: 96.6% female	GP: 2010 WM: 2317	Subscale inter-correlations Internal consistency	BMI (-) Eating disinhibition (-) Eating for pleasure (+/-) Eating restraint (+/-) Emotional overeating (-) Hunger susceptibility (-) Positive emotional eating (+/-)
Demïrgül & Rïgó [49] *Turkey*	General population	$\bar{x}$ = 29.3 yrs	35.8% female	159	Mean scale scores Internal consistency	Orthorexia (-) Emotion dysregulation (-)
Faw et al. [50] *USA* *White/Caucasian*	Female university students	$\bar{x}$ = 18.9 yrs	100% female	309	Mean scale scores Internal consistency	Body beliefs (-) Corumination (-) Disordered eating (-) Relationship satisfaction (+) Weight-contingent self-worth (-)
Fitch et al. [51] *UK*	General population	Study 1: M $\bar{x}$ = 29.4, F $\bar{x}$ = 26.89 yrs Study 2: M $\bar{x}$ = 29.37 yrs	Study 1: 50% female Study 2: 100% male	Study 1: 90 Study 2: 123	Mean scale scores Sex differences	Body image (+) Self-compassion (+) Weight (-)
Frazier and Bazo Perez [52] *USA* *White*	Women aged 40–65 who are biologically able to experience menopause	$\bar{x}$ = 52.63 yrs (range of 40-65 years)	100% female	467	Mean scale scores	Body dissatisfaction (-) Menopause symptoms (-) Positive perception of aging (+) Social support (-)
Gan & Yeoh [53] *Malaysia* *Chinese*	University students	$\bar{x}$ = 20.9 yrs (range of 18–25 yrs)	78.7% female	333	Sex differences Internal consistency	Body acceptance by others (+) Body appreciation (+) Body function (+) Disordered eating (-) General acceptance by others (+) Self-esteem (+)
Ge et al. [54] *USA*	General population	$\bar{x}$ = 44.35 yrs (range of 22–79 yrs)	53.2% female	941	Sex differences Internal consistency Dimensionality	Honoring feelings of hunger/satiety (+) Availability of healthy foods (+) Encouragement of diet diversity (+)
Gödde et al. [55] *Canada* *White/Caucasian*	General population, non-pregnant with no medical issues	$\bar{x}$ = 46.6 yrs	52% female	1466	Mean scale scores Sex differences Internal consistency	Cognitive eating restraint (-) Disordered eating (-) Self-esteem (+) Sociocultural pressure (-) Weight concern (-)
Green and García [56] *USA* *White/Caucasian*	Adults in rural communities	$\bar{x}$ = 32.21 yrs (range of 18-76 yrs)	78.6% female	187	Mean scale scores Sex differences Internal consistency	Anxiety (-) Depression (-) Self-esteem (+) Stress (-)
Haley et al. [57] *USA* *White/Caucasian*	Female university students & staff with BMI ≤ 25	$\bar{x}$ = 34.7 yrs (range of 18–59 yrs)	100% female	15	Mean scale scores Internal consistency	–
Henry et al. [22] *Australia*	Adults with severe mental illness	$\bar{x}$ = 42.1 yrs (range of 21-70 yrs)	54% female	122	Mean scale scores Internal consistency	BMI (-) Psychological distress (-)
Holmes et al. [58] *USA* *Black/African American*	Adult women who had experienced intimate partner violence	$\bar{x}$ = 34.91 yrs (range of 18-65 yrs)	100% female	216	Mean scale scores Internal consistency	Binge eating (-) Compensatory behaviour (-) PTSD symptoms (-)
Humphrey et al. [59] *USA* *White/Caucasian*	College students	Grp 1: $\bar{x}$ = 19 yrs, Grp 2: $\bar{x}$ = 19 yrs, Grp 3: $\bar{x}$ = 23 yrs	73.2% female	157	Mean scale scores	–
Jackson et al. [60] *USA* *White/Caucasian*	General population	$\bar{x}$ = 46 yrs	51.1% female	351	Mean scale scores Internal consistency	–
Jackson et al. [61] *USA* *White/Caucasian*	General population	$\bar{x}$ = 47.3 yrs	51.1% female	305	Mean scale scores Sex differences Internal consistency Dimensionality	Added sugar intake (+/-) Fruit intake (+) Vegetable intake (+/-) Whole grains intake (+)
Jackson et al. [62] *USA* *White/Caucasian*	General population	$\bar{x}$ = 45.19 yrs	65.3% female	49	Mean scale scores Internal consistency	–
Jackson et al. [63] *USA* *White/Caucasian*	General population	$\bar{x}$ = 47.88 yrs	51.7% female	288	Mean scale scores Subscale inter-correlations Internal consistency	Body appreciation (+) Functionality appreciation (+) Body shape preoccupation (-) Uncontrolled eating (-) Cognitive restraint (+/-) Emotional eating (-) Diet quality (+/-)
Jeune et al. [64] *USA* *White/Caucasian*	Non-nutrition major undergraduate college students	$\bar{x}$ = 23.4 yrs	100% female	229	Internal consistency	Interoceptive awareness (+) Body responsiveness (+) Self-regulation of eating (+) Emotional eating (-) External eating (-) Uncontrolled eating (-) Cognitive restraint (-)
Jordan and Musher-Eizenman [65] *USA* *White/Caucasian*	Undergraduate women (UG) and their mothers (M)	UG: $\bar{x}$ = 19.5 yrs M: $\bar{x}$ = 48.8 yrs	100% female	UG: 248 M: 138	Mean scale scores Internal consistency	BMI (-) Body appreciation (+) Eating pathology (-) Negative body talk (-) Positive body talk (+)
Jospe et al. [66] *New Zealand* *European*	Dunedin (NZ) residents with a BMI > 27 but without CV or metabolic disease	$\bar{x}$ = 40.8 yrs	62% female	50	Mean scale scores	–
Katcher et al. [67]	Undergraduate students with high dietary restraint and low body appreciation	$\bar{x}$ = 20.9 yrs (range of 19-26 yrs)	100% female	14	Internal consistency	–
Keirns & Hawkins [68] *White/Caucasian*	University students/General population enrolled in larger weight-loss trial	$\bar{x}$ = 32.2 yrs	73.4% female	248	Mean scale scores Internal consistency	–
Keirns & Hawkins [69] *White/Caucasian*	University students/General population enrolled in larger weight-loss trial	$\bar{x}$ = 34.4 yrs	72.9% female	136	Mean scale scores Internal consistency	Body concern (-)
Kelly & Stephen [70] *Canada* *White/Caucasian*	Undergraduate students	$\bar{x}$ = 19.7 yrs	100% female	92	Mean scale scores Internal consistency	Body appreciation (+) Body image (+) Body satisfaction (+) Dietary restraint (-) Self-compassion (+) Self-esteem (+)
Khalsa et al. [71] *USA* *Black/Afr. American*	Parents of infants, predominantly low-income	$\bar{x}$ = 27 yrs (of parents)	90% female	204	Dimensionality	–
Khalsa et al. [72] *USA* *Black/Afr. American*	Parents with children aged between 5.5 and 12.5 months	$\bar{x}$ = 27.2 yrs (of parents)	91% female	201	Mean scale scores Subscale inter-correlations Internal consistency	Infant feeding style (+/-)
Koller et al. [73] *USA*	Women with eating disorders & a control group	–	100% female	97	Mean scale scores Internal consistency	–
Linardon & Mitchell [15] *Australian*	General population	M $\bar{x}$ = 25.4, F $\bar{x}$ = 24.8 yrs (range of 18–65 yrs)	80.3% female	375	Mean scale scores Internal consistency	Body appreciation (+) Body checking (-) Binge eating frequency (-) Dichotomous thinking (-) Disinhibited eating (-) Exercise motivation (-) Rigid dietary control (-) Weight/shape overevaluation (-)
Linardon et al. [74] *Australia* *White/Caucasian*	Australian women	$\bar{x}$ = 29.43 yrs	100% female	1000	Mean scale scores Internal consistency	Clinical impairment (-) Disordered eating (-) Flexible eating control (-) Inflexible eating (-) Rigid eating control (-)
Linardon et al. [75]	General population	$\bar{x}$ = 29.2 yrs	91% female	1341	Mean scale scores Internal consistency	BMI (-) Dichotomous thinking (-) Flexible dietary control (-) Inflexible eating beliefs (-) Rigid dietary control (-)
Linardon [76] *White/Caucasian*	General population	$\bar{x}$ = 32.33	100% female	3039	Mean scale scores Internal consistency	Body appreciation (+) Functionality appreciation (+) Body image flexibility (+)
Liu et al. [77] *White/Caucasian*	General population	$\bar{x}$ = 32.3 yrs (range of 18-71 yrs)	100% female	1260	Internal consistency	Binge eating (-) Body dissatisfaction (-) Driven exercise (-) Eating concern (-) Eating restraint (-) Emotion regulation difficulties (-) Overvaluation of shape/weight (-) Self-compassion (+)
Loor et al. [78] *Hispanic*	General population	$\bar{x}$ = 24.32 yrs (range of 18-57 yrs)	87.5% female	104	Mean scale scores Internal consistency	Mood (+) Guilt after eating (-) Anticipated enjoyment of taste (+) Eating restraint (-)
Lopez et al. [79] *USA* *Black/Afr. American*	Undergraduate students	18-24 yrs: 92%, 25 + yrs: 8%	44% female	758	Mean scale scores Subscale inter-correlations Internal consistency	–
Loughran et al. [80] *USA* *White/Caucasian*	University students	–	85% female	146	Mean scale scores Internal consistency	–
Lovan et al. [81] *USA* *White/Caucasian*	Undergraduate students	$\bar{x}$ = 19.8 yrs	62.1% female	66	Subscale inter-correlations Sex differences Internal consistency	BMI (-) Body awareness (+) Interoceptive responsiveness (+) Emotional eating (-) Uncontrolled eating (-) Cognitive restraint (+/-) Restrained eating (-) External eating (+/-)
Martin-Wagar & Heppner [19] *USA* *White/Caucasian*	Patients at an eating disorder treatment centre	$\bar{x}$ = 31.97 yrs (range of 18-67 yrs)	92% female	224	Mean scale scores Subscale inter-correlations Internal consistency Dimensionality	Eating pathology (-) Clinical impairment (-) Body image (-) Depression (-) Cognitive flexibility (+) Gastrointestinal symptoms (+/-)
Messer et al. [82] *White/Caucasian*	General population	–	–	3039	Mean scale scores Internal consistency	Disordered eating (-)
Miller et al. [83] *Canada* *White/Caucasian*	Female undergraduates	$\bar{x}$ = 19.7 yrs	100% female	92	Mean scale scores Internal consistency	Body appreciation (+) Body satisfaction (+) Dietary restraint (-)
Modica [84] *USA* *White/Caucasian*	Adults who had people they considered to be family	$\bar{x}$ = 45.03 yrs (range of 30-70 yrs)	50% female	522	Mean scale scores Subscale inter-correlations Sex differences Internal consistency	Body appreciation (+) Internal body orientation (+)
Modica & DiLillo [85] *USA* *White/Caucasian*	Cisgender women	$\bar{x}$ = 24.17 yrs (range of 18-30 yrs)	100% female	1275	Mean scale scores Subscale inter-correlations Internal consistency	BMI (-) Unconditional acceptance (+) Body acceptance (+) Functionality appreciation (+) Body appreciation (+)
Morris et al. [86] *USA* *White/Caucasian*	Cisgender college students	$\bar{x}$ = 19.97 yrs	70.4% female	345	Mean scale scores Sex differences	Eating pathology (-)
Munroe et al. [87] *White/Caucasian*	General population	$\bar{x}$ = 34.87 yrs	100% female	278	Mean scale scores Subscale inter-correlations Internal consistency	Embodiment (+) Life satisfaction (+/-) Negative affect (-) Pessimism (-) Self-compassion (+)
Murray et al. [88] *White/Caucasian*	General population	$\bar{x}$ = 39.26 yrs (range of 18-74 yrs)	49.4% female	498	Mean scale scores Subscale inter-correlations Sex differences Internal consistency	Body surveillance (-) Aesthetic investment (+/-) Aesthetic satisfaction (+) Functional investment (+/-) Functional satisfaction (+) Functional appreciation (+) Body appreciation (+) BMI (-)
Nelson et al. [24] *USA White/Caucasian*	Adolescents in eating disorder treatment and their caregivers	Patients: $\bar{x}$ = 15.14 yrs (range of 11–18 yrs)	Patients: 87.2% female	47 pairs (child/ Caregiver)	Mean scale scores Subscale inter-correlations Internal consistency	Weight change (+) Dietary restraint (-) Eating concern (-) Shape concern (-) Weight concern (-) Eating disorder symptoms (-) Psychosocial impairment (-)
Palascha et al. [89] *UK & USA*	General population	18-24 yrs: 13.2%, 25–34yrs: 19.6%, 35–44 yrs: 21.6%, 45–54yrs: 23.6%, 55–65 yrs: 22%	53.7% female	2174	Internal consistency	Binge eating (-) BMI (-) Body appreciation (+) Eating competence (+) Internally regulated eating (+) Proactive coping (+) Restrictive eating (-) Satiety responsiveness (+) Satisfaction with life (+) Self-esteem (+) Slowness in eating (+) Weight change (-) Weight cycling severity (-)
Parsons et al. [90] *White/Caucasian*	Male sexual minority couples in a married relationship	$\bar{x}$ = 52.58 yrs	0% female	456	Dimensionality	–
Patel et al. [91] *UK* *White/Caucasian*	Adults with a BMI > 25	$\bar{x}$ = 47.3 yrs (range of 19-72 yrs)	83% female	18	Mean scale scores	–
Peschel et al. [92] *USA*	Female university students	$\bar{x}$ = 19.64 yrs	100% female	39	Mean scale scores	–
Rodgers et al. [93] *USA*	Undergraduate students	$\bar{x}$ = 19.84 yrs (range of 18-25 yrs)	81% female	605	Mean scale scores Subscale inter-correlations Internal consistency	Eating competence (+) Orthorexia behaviours (-)
Rodgers et al. [94] *White/Caucasian*	Women aged 60–75	$\bar{x}$ = 65.2 yrs	100% female	171	Mean scale scores Internal consistency	Appearance satisfaction (+) Body appreciation (+) Body image-related QoL (+) Depression (-) Positive reappraisal/acceptance of appearance changes (+)
Rogers et al. [95] *White/Caucasian*	Women with elevated body image dissatisfaction/lower body appreciation	$\bar{x}$ = 20.1 yrs	100% female	30	Mean scale scores Internal consistency	–
Romano et al. [96] *USA* *White/Caucasian*	College students	$\bar{x}$ = 24.4 yrs	68.2% female	902	Internal consistency	–
Romano & Heron [97] *USA* *White/Caucasian*	College students	$\bar{x}$ = 22.27 yrs	75.81% female	1228	Internal consistency	–
Sandler et al. [98] *Canada* *White/Caucasian*	Men aged 18–30	$\bar{x}$ = 23.2 yrs (range of 18-30 yrs)	0% female	249	Mean scale scores Internal consistency	Self-esteem (+) Fear of self-compassion (-) Body parts satisfaction (+) Body (self)-compassion (+) Conformity to masculine norms (+)
Sarcona et al. [20] *USA* *Black/African American*	Adults with chronic kidney disease on haemodialysis	18-64 yrs: n = 32 (49%) >65yrs: n = 33 (51%)	49% female	65	Mean scale scores Sex differences	–
Saunders et al. [79] *USA* *Cuban*	College students	$\bar{x}$ = 21.4 yrs (range of 18-53 yrs)	77% female	482	Internal consistency Dimensionality	–
Schmid et al. [99] *USA* *White/Caucasian*	University employees enrolled in the Nourish Your Whole Self programme	$\bar{x}$ = 47.0 yrs	91.2% female	114	Mean scale scores	–
Schueler et al. [100] *USA* *White/Caucasian*	Undergraduate students	Range of 18–25 yrs	57.9% female	299	Mean scale scores Internal consistency	–
Smith et al. [101] *USA* *White/Caucasian*	College students	$\bar{x}$ = 21 yrs	74% female	478	Mean scale scores Subscale inter-correlations Sex differences Internal consistency	BMI (-) Disordered eating (-) Emotional eating (-)
Soulliard & Vander Wal [102] *White/Caucasian*	Sexual orientation minority adults	$\bar{x}$ = 32.5 yrs (range of 18-65 yrs)	53.4% female	223	Mean scale scores Internal consistency	Body appreciation (+) Body image acceptance (+) BMI (-) Functionality appreciation (+)
Soulliard et al. [21] *USA* *White/Caucasian*	Trans or non-binary adults	$\bar{x}$ = 24.7 yrs	8 gender categories; largest was trans man (33.1%)	148	Mean scale scores Internal consistency	Body appreciation (+) Non-affirmation of gender identity (-)
Spoor & Madanat [103] *USA* *White/Caucasian*	First year female undergraduate students	–	100% female	44	Mean scale scores Internal consistency	–
Swami et al. [104] *USA* *White/Caucasian*	General population	$\bar{x}$ = 37.7 yrs (range of 18-75 yrs)	50.3% female	603	Subscale inter-correlations Construct validity Internal consistency Dimensionality	BMI (+/-) Body acceptance by others (+) Body appreciation (+/-) Self-esteem (+/-)
Tabatabai et al. [105] *USA* *White/Caucasian*	College students	$\bar{x}$ = 23.5 yrs (range of 18-56 yrs)	75.2% female	307	–	Diet quality (+/-) Chips (crisps) consumption (-) Fast food consumption (-)
Teas et al. [23] *USA*	Community older adults	$\bar{x}$ = 68.8 yrs (range of 58-83 yrs)	72% female	79	Mean scale scores Internal consistency	BMI (-) Intrinsic exercise motivation (-) Triglycerides (+) LDL/HDL ratio (+) C-reactive proteins (+)
Tylka & Wood-Barcalow [106] *USA* *White/Caucasian*	Psychology students	$\bar{x}$ = 20.3 yrs (range of 18-56 yrs)	54.4% female	675	Mean scale scores Internal consistency	Appearance evaluation (+) Body appreciation (+) Body dissatisfaction (-) Body surveillance (-) Disordered eating (-) Internalisation of societal appearance standards (-) Proactive coping (+) Self-esteem (+)
Tylka et al. [14] *USA* *White/Caucasian*	General population	$\bar{x}$ = 33.8 yrs	50.26% female	385	Mean scale scores Internal consistency	Binge eating (-) BMI (-) Body appreciation (+) Flexible dietary control (-) Food preoccupation (-) Poor interoceptive awareness (-) Life satisfaction (+) Positive (+) & negative (-) affect Rigid dietary control (-)
Tylka et al. [107] *USA* *White/Caucasian*	General population (only Studies 2 & 4 used IES-2)	Study 2: $\bar{x}$ = 34.75 yrs (range of 18–74 yrs) Study 4: $\bar{x}$ = 33.76 yrs (range of 18–75 yrs)	Study 2: 48.2% female Study 4: 47.1% female	Study 2: 253 Study 4: 382	Internal consistency	Body appreciation (+) Appearance evaluation (+) Functionality appreciation (+) Body image flexibility (+) Body surveillance (-) Thin-ideal internalisation (-) Muscular-ideal internalisation (-) Gratitude (+) Negative affect (-) BMI (-) Dietary restriction (+/-) Binge eating (+/-) Food preoccupation (-)
Virani et al. [108] *USA* *White/Caucasian*	Bariatric surgery patients	$\bar{x}$ = 49.6 yrs (range of 23-75 yrs)	77.8% female	90	Mean scale scores	Emotional eating (-)
Voelker et al. [18] *USA* *White/Caucasian*	Patients receiving treatment for an eating disorder	Not given	89.1% female	165	Mean scale scores Internal consistency	Body intuition (+) Exercise variety (-)
Webb & Hardin [109] *USA* *White/Caucasian*	Undergraduate women	$\bar{x}$ = 19.4 yrs (range of 18-27 yrs)	100% female	362	Mean scale scores Internal consistency	BMI (-) Body image flexibility (+) Body shame (-) Internalised weight bias (-) Self-compassion (+)
Yoon et al. [110] *USA* *Asian American*	Undergraduate students	$\bar{x}$ = 20.9 yrs	54.5% female	828	Internal consistency	–

Key: (+) indicates positive association, (-) indicates negative association. UPE = Unconditional permission to eat subscale; EPR = Eating for physical rather than emotional reasons subscale; RHSC = Reliance on hunger and satiety cues subscale; BFCC = Body-food choice congruence subscale.

### 4.4. Mean IES-2 scores

Forty out of 90 studies (44.4%) provided mean scores for the IES-2 total and each of the four IES-2 subscales. A further 37 studies (41.1%) provided mean scores for just the IES-2 total and two more studies provided mean scores for just the IES-2 subscales. Highest and lowest scores for the IES-2 total and all subscales are provided in Table 3. Notably, the studies that account for many of the results used clinical samples of people who are either of a higher weight or who have a current or historical eating disorder. In fact, whilst the vast majority of mean scores fell between 3.00 and 3.99, those studies with mean scores below 3 (demonstrating that participants did not generally engage in intuitive eating behaviours) were almost all within people of a higher weight or within eating disorder or disordered eating populations. If the 17 studies that are focused on weight or eating disorders [18,19,24,31–33,35,38,41,44,45,47,66,73,91,69,111] are not taken into account, the sample characteristics are slightly more varied and scores tend more towards the centre of the scale’s 1–5 range (see the second part of Table 3). There were very few scores that fell at 4.00 or above and no commonalities were identified between populations where this occurred.

**Table 3:** Highest and lowest mean scores for IES-2 total and subscales.

	Lowest		Highest	
Variable	Mean (SD)	Sample characteristics	Mean (SD)	Sample characteristics
***Full sample***				
IES-2 total	2.12 (0.32)	People with Binge Eating Disorder [19]	4.04 (0.54)	General population [42]
UPE	2.04 (0.34)	USA general population [62]	4.0 (0.58)	Post-HAES intervention [59] Men without orthorexia [32]
EPR	1.61 (1.07)	People with Binge Eating Disorder [19]	4.50 (0.45)	Women in not-stable ED recovery [73]
RHSC	1.79 (0.67)	People with Bulimia Nervosa [33]	4.1 (0.53)	Post-HAES intervention [59]
BFCC	2.51 (0.93)	People with Binge Eating Disorder [19]	4.50 (0.72)	Women in stable ED recovery [73]
***Excluding studies focused on weight or eating disorders***				
IES-2 total	2.75 (0.56)	Pre-self-compassion intervention [57]	4.04 (0.54)	General population [42]
UPE	2.04 (0.34)	USA general population [62]	4.0 (0.58)	Post-HAES intervention [59]
EPR	2.84 (0.91)	Sexual orientation minority women [102]	3.9 (0.8)	Male college students [86]
RHSC	2.95 (0.87)	Sexual orientation minority women [102]	4.1 (0.53)	Post-HAES intervention [59]
BFCC	3.09 (0.89)	Sexual orientation minority women [102]	3.8 (0.64)	Post-HAES intervention [59] Students with intermittent fasting behaviours [100]

Numbers in square brackets are citations. IES-2 = Intuitive Eating Scale-2; UPE = Unconditional permission to eat subscale; EPR = Eating for physical rather than emotional reasons subscale; RHSC = Reliance on hunger and satiety cues subscale; BFCC = Body-food choice congruence subscale; ED = Eating disorder; HAES = Health at Every Size.

### 4.5. IES-2 inter-correlations

Eleven out of 90 studies (12.2%) provided correlation estimates between total IES-2 scores and the four subscales. A further six studies provided correlation estimates between just the four subscales. Statistically significant inter-correlations ranged between −0.49 and 0.92; see Table 4 for the highest and lowest statistically significant correlations between IES-2 total and subscale scores. Correlations that were not statistically significant are not presented.

**Table 4:** Highest and lowest correlations between IES-2 total and subscale scores.

Variable		1	2	3	4	5
1.	Total intuitive eating	–	0.66** [24]	0.92** [84]	0.89** [24]	0.61** [24,84]
2.	Unconditional Permission to Eat	0.28* [72]	–	0.28** [84]	0.64** [24]	0.40** [24]
3.	Eating for Physical… Reasons	0.64*** [35]	−0.49** [19]	–	0.66** [84]	0.51** [84]
4.	Reliance on Hunger/Satiety Cues	0.68** [85]	0.06** [48]	0.14* [87]	–	0.61** [47,87]
5.	Body-Food Choice Congruence	0.34*^(^*^)^ [72,112]	−0.45* [81]	0.17* [72]	−0.30** [63]	–

Numbers in square brackets are citations. Highest correlations are above the diagonal, lowest are below. * *p* < .05, ** *p < .01,* *** *p* < .001. Bracketed asterisks indicate differing statistical significance levels between studies.

### 4.6. Construct validity

The review found a large amount of evidence for the convergent construct validity of the IES-2, in terms of it being associated with measures of constructs that would be expected to be related to intuitive eating e.g., [113–116]. Twenty-one out of 90 studies found the IES-2 to be negatively associated with BMI, although, interestingly, Tylka et al. [14] also found a small-to-medium positive association (*r* = 0.28, *p* < .001) between the IES-2 and BMI in their subsample of male participants. Several studies found the IES-2 to be positively associated with body appreciation, self-compassion and self-esteem and negatively associated with depression and anxiety, disordered eating behaviour/eating disorder symptomology and rigid dietary control. Intuitive eating as measured by the IES-2 appeared in general to not be statistically significantly related to age [14,51,88,109,40,75], other than in one study of older adults [23] which found a moderate association (*r* = 0.32, *p* < .01).

A full list of all constructs that were statistically significantly associated with the IES-2 can be found in the S2 Table. None of the studies included in the review provided evidence for the discriminant construct validity of the scale.

### 4.7. Sex differences

Thirteen out of 90 studies tested for sex differences in levels of intuitive eating. Of these, six found statistically significant differences between total IES-2 scores for men and women, with men consistently scoring more highly [51,84,88,101,55,61]. Effect sizes ranged from *d* = 0.28 [84] to *d* = 0.42 [88] across the five studies that reported them. One study found statistically significant sex differences in ‘Unconditional permission to eat’ subscale scores [101], with men again scoring more highly and a reported effect size of *d* = 0.30. Seven studies found statistically significant sex differences in ‘Eating for physical rather than emotional reasons’ subscale scores [54,81,84,86,88,101,61], with men consistently scoring more highly. Effect sizes ranged from *d* = 0.29 [84] to *d* = 0.80 [101] across the four studies that reported them (although the latter effect size should be interpreted with caution as this is far higher than would be expected). Two studies found statistically significant sex differences in ‘Reliance on hunger and satiety cues’ subscale scores [84,88], with men again scoring more highly and reported effect sizes between *d* = 0.29 [84] and *d* = 0.31 [88]. Murray et al. [88] also found statistically significant sex differences in ‘Body-food choice congruence’ subscale scores, with men again scoring higher than women and a reported effect size of *d* = 0.22.

### 4.8. Reliability

The COSMIN methodology for systematic reviews of patient-reported outcome measures [117] recommend that internal consistency (Cronbach’s alpha (α) or McDonald’s omega (Ω)) should be at least 0.7. Out of 90 studies, 67 reported an internal consistency statistic for the IES-2 overall, with 66 reporting an internal consistency statistic of 0.7 or higher. Twenty-nine studies provided internal consistency statistics for the ‘Unconditional permission to eat’ subscale, with 21 reporting alphas of 0.7 or higher. Twenty-eight studies reported on the internal consistency of the ‘Eating for physical rather than emotional reasons’ subscale, all of which were 0.7 or higher. Twenty-eight studies reported on the internal consistency of the ‘Reliance on hunger and satiety cues’ subscale, with all but one of these reporting alphas of 0.7 or higher. Finally, 30 studies reported on the internal consistency of the ‘Body-food choice congruence’ subscale, with all but one of these reporting alphas of 0.7 or higher.

None of the studies included in the review provided evidence for any other types of reliability (e.g., test-retest or split half).

### 4.9. Dimensionality

Ten studies reported on the factor structure of the IES-2. Five of these [19,33,36,90,104] tested the factor structure found in the original development and validation paper for the IES-2 [2] of four latent factors loaded onto a higher order intuitive eating factor. Babbott et al. [33] found that, in their sample of people seeking eating disorder treatment, this model had negative residual variance, suggesting that the model was misspecified. Swami et al. [104] found this model to be a reasonable-to-good fit to the data in their general population sample (CFI = .96, TLI = .93, RMSEA = .08, SRMR = .03) but only when the variances between the negatively worded items were allowed to correlate. Martin-Wagar and Heppner [19], Parsons et al. [90] and Bazo Perez et al. [36] found this model to be a poor fit to the data in their samples of eating disorder patients (CFI = 0.89, TLI = 0.87, RMSEA = 0.10, SRMR = 0.10), married male sexual minority couples (CFI = 0.69, TLI = 0.67, RMSEA = 0.10, SRMR = 0.13) and Hispanic-majority young adults (CFI = 0.79, TLI = 0.76, RMSEA = 0.13, SRMR = 0.13) respectively.

Six studies [33,37,54,79,61,71] tested a factor structure of four latent factors with the 23 IES-2 items as indicators but no hierarchical intuitive eating factor. In Belon et al.’s [37] sample of college women, this model showed an acceptable fit to the data, with the exception of the RMSEA (CFI = .92; TLI = .91; RMSEA = .10; SRMR = .08). In Jackson et al.’s [61] general population sample this model fitted well (CFI = .98, TLI = .98, SRMR = .05, RMSEA = .04) but only with modifications (two items were removed as their factor loadings were less than 0.5 and errors with modification indices greater than 10 that were theoretically similar were allowed to correlate). In Babbott, Mitchison, et al.’s [33] sample of people seeking eating disorder treatment, this model did not fit unless co-variances were added to similarly phrased items (22 & 23, 13 & 14, 7 & 8), after which it showed acceptable fit to the data (CFI = .93, SRMR = .10, RMSEA = .07). Both Khalsa et al. [71] in their sample of racial minority and/or low-income parents and Saunders et al. [79] in their sample of Hispanic American college students found this model to be an inadequate fit to the data. Ge et al. [54] stated that this model showed acceptable fit to the data in their general population sample (CFI = 0.90, TLI = 0.88, RMSEA = 0.08, SRMR = 0.09) although arguably the TLI and SRMR estimates suggest less-than-adequate model fit. Finally, Swami et al. [104] also tested a bifactor model which contained four factors with individual items as indicators as well as an overall intuitive eating factor with individual items as indicators. This gave the best fit to the data in their general population sample (CFI = .97, TLI = .94, RMSEA = .08, SRMR = .02), again with correlation allowed between the variances of the negatively worded items.

Four studies performed subsequent exploratory factor analyses after testing the original factor structure and finding it to be a poor fit to their sample [19,79,90,71]. In their sample of eating disorder patients, Martin-Wagar and Heppner [19] found a four-factor, 22-item model fit their data best. This utilised the original four factors found by Tylka and Kroon Van Diest [2] but with the items distributed differently across the ‘Unconditional permission to eat’ and ‘Eating for physical rather than emotional reasons’ factors and with item 11 removed due to low factor loading. They also found that those scoring highly on most of the items in the ‘Eating for physical rather than emotional reasons’ factor were low-scoring on items 4 and 5 (“If I am craving a certain food, I allow myself to have it” and “I allow myself to eat what food I desire at the moment”), suggesting that therefore these items should be reverse-scored in eating disorder samples. Parsons et al. [90] found that, in their sample of married male sexual minority couples, a five-factor, 23-item model fit their data the best, utilising the original four IES-2 factors with an additional factor of ‘Eating not as coping’. In their sample of racial minority and/or low income parents, Khalsa et al. [71] found that a six-factor, 23-item model fit their data best, although a subsequent confirmatory factor analysis on a novel sample was not performed. This model utilised all of the items in the IES-2 but arranged them into the factors of (1) Avoiding forbidden foods; (2) Permission to eat; (3) Avoiding emotional eating; (4) Avoiding food-related coping strategies; (5) Reliance on hunger and satiety cues; and (6) Body-food choice congruence. Finally, Saunders et al. [79] found that in their sample of Hispanic American college students, a three-factor, 11-item model fit their data best. This was achieved by splitting their dataset and performing an exploratory factor analysis on one subsample, and then performing a subsequent confirmatory factor analysis on the other subsample. This model excluded the ‘Unconditional permission to eat’ subscale and retained the other three subscales from the original scale, and was found to have excellent fit to their data (CFI = 0.99, TLI = 0.98, RMSEA = 0.05). This model was also tested by Bazo Perez et al. [36] in their Hispanic-majority sample of young adults, and was similarly found to fit their data well (CFI = 0.91, TLI = 0.93, RMSEA = 0.08, SRMR = 0.08).

## 5. Discussion

The aim of this study was to assess the psychometric properties of the IES-2 across diverse populations and contexts, and to ascertain how it is related to other measures of physical and psychological health. In total, 90 studies were included in the review, covering a range of samples including college students, people from the general population/community, and people seeking treatment for eating disorders or weight management. Studies that reported the country in which the research took place were predominantly from the USA but also included Australia and New Zealand, the UK, Canada, Malaysia and Turkey. Mean age of participants ranged from 15.14 to 68.8 years, and in most studies, women accounted for more than 50% of participants, with 24 studies recruiting only women. The majority of studies in the review reported samples that were predominantly white or Caucasian, although five studies did report a majority Black or African American sample and two reported a majority Hispanic sample.

### 5.1. Psychometric properties

The vast majority of studies that reported the internal consistency of the overall IES-2 and/or its subscales achieved the cut-off of 0.7 or higher recommended by the COSMIN methodology [117], although it is notable that a smaller proportion of studies met this criteria for the ‘Unconditional permission to eat’ subscale as compared to overall IES-2 scores and the other three subscales. These findings support the internal consistency reliability of the IES-2 and at least three of the four subscales. Unfortunately, none of the studies included in the review provided evidence for any other types of reliability of the IES-2 (such as test-retest or split half).

Of the 90 studies included in the review, 78 reported mean scores for either the total IES-2 or at least one of the subscales. In a five-point scale such as that used for IES-2 responses, mean scores would be expected to fall within the 2−4 range [118]. When all studies were included in the analysis, this was the case for ‘Unconditional permission to eat’ scores but not for total IES-2 scores or the ‘Eating for physical rather than emotional reasons’, ‘Reliance on hunger and satiety cues’ and ‘Body-food choice congruence’ subscales. However, when the 17 studies that were focused on weight or eating disorders were excluded, mean scores tended more towards the centre. In this context, only total IES-2 scores and the ‘Reliance on hunger and satiety cues’ subscale fell outside of the 2−4 range, and even this was only slightly (with a highest mean score of 4.04 for the IES-2 total and 4.1 for the ‘Reliance on hunger and satiety cues’ subscale). This suggests that in samples focused on weight or eating disorders, mean scores may tend more towards the extremes of the scale.

Convergent construct validity of the scale was well supported, as the IES-2 was found to be negatively associated with several constructs it would be expected to, including BMI, depression and anxiety, disordered eating/eating disorder symptomology and rigid dietary control. Similarly, it was also positively associated with body appreciation, self-compassion and self-esteem. Unfortunately, none of the studies included in the review provided evidence for the IES-2’s discriminant construct validity.

In terms of known-groups construct validity, 13 studies tested for sex differences in intuitive eating scores and six found statistically significant differences in total IES-2 scores, with men scoring higher. Men have generally been found to have higher levels of intuitive eating than women [e.g., 2] so this supports the known-groups construct validity of the scale, i.e., that certain groups of people might be expected to score differently to others and therefore the scale should be sensitive enough to detect this [119]. Seven of the 13 studies also reported sex differences in the ‘Eating for physical rather than emotional reasons’ subscale and differences were also found in the ‘Unconditional permission to eat’ and ‘Body-food choice congruence’ subscales (one study each, respectively), each time with men scoring more highly. Finally, two studies found statistically significant sex differences in the ‘Reliance on hunger and satiety cues subscale’, again with men scoring more highly. These findings show a consistent pattern of the IES-2 behaving as it should do, both in terms of its association with constructs that it *should* have an association with and in terms of it detecting known sex differences in intuitive eating levels. This suggests that the instrument is indeed measuring the construct that it purports to measure, i.e., intuitive eating [115].

### 5.2. Dimensionality

Inter-correlations between total IES-2 scores and its subscales were reported in 11 out of the 90 studies in the review. Correlations between the subscales and total IES-2 scores were mixed, but were particularly low for the ‘Unconditional permission to eat’ and ‘Body-food choice congruence’ subscales. Both of these subscales also correlated negatively with some of the other subscales. This suggests that the ‘Unconditional permission to eat’ and ‘Body-food choice congruence’ subscales may be less strongly related to overall intuitive eating than the other subscales.

Ten studies reported on the factor structure of the IES-2. Five tested the original factor structure of four factors with a hierarchical intuitive eating factor, which was not promising in terms of fit to the data in any of the studies. Six tested a four-factor model without a hierarchical intuitive eating factor, but only one study found this model to have acceptable fit [37]; notably, this was using a sample of college women and therefore was very similar to the samples in which the four-factor conceptualisation of the IES-2 was originally developed and validated in.

Three of the studies that did not find the four-factor model to fit at all were using predominantly non-white samples [36,79,71], which suggests that the construct of intuitive eating as represented by Tylka and Kroon Van Diest’s [2] four-factor model may possibly be representative of Western or Caucasian eating styles that don’t necessarily reflect what is considered normal, healthy eating in other cultures [104]. This is in keeping with the findings of other studies [e.g., 43], who have also highlighted differences in intuitive eating according to socioeconomic status and race.

Four studies performed exploratory factor analyses [19,79,90,71] to discover whether there was a factor structure that represented their data better. All found an alternative factor structure that fit their data better than the factor structured posited in the original development and validation paper; two of these retained all 23 of the IES-2 items but arranged them into an alternative factor structure, one retained 22 of the items but reverse-scored two of the remaining items and arranged them into a four-factor structure, and the other only retained 11 of the original IES-2 items and arranged them into a three-factor model. Interestingly, this latter study, which was conducted in a Hispanic-majority sample, excluded the ‘Unconditional permission to eat’ subscale and its items, which this review has highlighted as being potentially problematic from a psychometric perspective. This factor structure has also recently been validated in another Hispanic-majority sample [36], suggesting that the original factor structure of the IES-2 may not perform as well in samples that differ from the Western college student sample that the scale was developed in, and that the ‘Unconditional permission to eat’ subscale in particular may not be as applicable to other samples or may be especially sensitive to cultural differences in eating styles or other contextual factors.

### 5.3. Strengths, limitations and future research directions

Although there have been systematic reviews that examine the psychosocial correlates of intuitive eating, this study is the first to specifically systematically review the psychometric performance of the IES-2 in its original English form since its publication twelve years ago. The review provides evidence for the measure’s internal consistency, as well as its convergent and known-groups construct validity, and has provided a comprehensive account of other psychological and physical health constructs with which it is associated. This review has also made novel contributions in identifying that studies relating to weight or eating disorders often report mean intuitive eating scores that tend towards the extremes of the scale, suggesting that studies of this kind may need to be considered separately in future reviews, particularly when statistical aggregation of scores or meta-analyses are being performed. This study has also identified that the four-factor structure of the IES-2 may not be representative of the construct of intuitive eating in samples that differ greatly from those in which the IES-2 was developed (i.e., Western college students).

One potential drawback to this review was that the samples of the studies included tended to be majority female and white, and mainly took place in the USA or other countries in the Global North. With the exception of gender distribution, this is likely a corollary of the study’s eligibility criteria, which stipulated that studies must be available in English to be included. In particular, the search process identified a considerable number of studies undertaken in France, Turkey and several South American and Middle Eastern countries that were excluded due to language but suggestive of a research corpus that this review was unable to access. It was also decided to exclude studies published in English but that used non-English translations of the IES-2 as it was felt that it would be difficult to discern between true cross-cultural variation and variation brought about by differences in translation processes and quality [27,28], particularly as some translations of the IES-2 do not even have the same number of items as the original English version [e.g., 120, 121]. However, future reviews would benefit from being conducted in languages other than English, incorporating translation of non-English texts to diversify the samples included, and including studies using non-English translations of the IES-2 in order to allow for the assessment of the scale’s cross-cultural validity. The authors also acknowledge that grey literature and non-indexed sources were not included in this review due to limited time and financial resources, and authors were not contacted regarding unpublished studies. As such, there is a risk of publication bias. However, this review does not report pooled effect size estimates; instead, it presents ranges across studies to reflect variability and provide a broader perspective on the findings. The authors agree that future reviews could benefit from including grey literature and non-indexed sources to further reduce the risk of bias and better capture the full scope of available evidence.

#### 5.3.1. Risk of bias.

The quality of each study included in this review was assessed using two domains from the COSMIN Risk of Bias checklist [25,26]. However, as noted in Section 4.2, a large number of the studies that were rated ‘Inadequate’ only received this rating because they did not provide an internal consistency statistic for the IES-2 and its subscales. The COSMIN Risk of Bias checklist is designed for systematic reviews of patient-reported outcome measures where the studies included are specifically focused on the measurement properties of an instrument. However, there was not a more appropriate risk of bias checklist than the COSMIN, and once the searches and quality assessment were concluded it became apparent that for studies that were only using the IES-2 as an outcome measure, the ratings in the COSMIN checklist for internal consistency were too harsh and this negatively affected the quality ratings of some of the studies. Specifically, another 15 studies would have received a ‘Very good’ rating if it had not been for this criterion. The authors are not aware of a more appropriate risk of bias checklist at the present time but recommend that similar reviews use a different approach than the COSMIN when considering study quality. It is acknowledged that the inclusion of papers that used the IES-2 as an outcome measure rather than only including papers that focused on the measurement properties of the instrument in line with COSMIN guidance could be considered to be a limitation of the review. However, there were very few papers that focused specifically on the measurement properties of the IES-2; specifically, there were only six papers that could be considered to meet this criteria in the first round of searches [37,61,79,104,71,33]. It was therefore felt that the review would be too limited if only these papers were included and that valuable psychometric information could be gained from studies that used it as an outcome measure.

#### 5.3.2. The Intuitive Eating Scale-3.

At time of writing, the Intuitive Eating Scale-3 has recently been published [16]. In their rationale for developing a third iteration of the scale, the authors state that the four-factor structure of the IES-2 often fails to replicate in both clinical and non-clinical samples, which is corroborated by the findings of this review. The new scale identifies four sub-domains of intuitive eating, which retain the same names as the IES-2 subscales, but all of the items from the IES-3 are novel, with no items retained from the IES-2. This means that it will not be possible to use the findings of this study to draw conclusions about the IES-3 as it is likely that the new items will perform differently to the IES-2 items from a psychometric perspective. Although the studies that formed the development of the IES-3 had a good balance of genders and ages, the samples were majority white, participants were all US citizens, and no clinical samples were used. Therefore, the IES-3’s performance in non-white, non-Western and clinical samples will be of particular interest.

Despite the publication of the IES-3, the current review remains highly significant and timely as it was the prevailing standard during the period under review and continues to be widely used in current research and practice. This review synthesises the available psychometric evidence, offering critical insight into the scale’s reliability, validity and applications across populations, and supporting users who are still employing the IES-2 due to the natural lag in adoption and availability of validation data for the newer version. This work also provides a necessary foundation for future comparative analyses with the IES-3, which has yet to accumulate sufficient empirical usage to support a systematic review. As such, this review not only documents the state of the evidence to date but also serves as a benchmark against which the utility of the new version can be assessed.

#### 5.3.3. Implications for research and practice.

There are a number of findings from this review that have implications for research and practice. Firstly, given that the four-factor model failed to replicate in a number of studies, it may be useful for studies that are intending to use the IES-2 as an outcome measure to confirm that this factor structure holds in their sample using confirmatory factor analysis or similar. Secondly, these results suggest that the factor structure of the IES-2 is sensitive to contextual factors such as cultural or racial differences. This means that findings should be interpreted cautiously if using the IES-2 in a non-Western or non-white sample and future research would benefit from refining and/or validating the scale in ethnically diverse, under-represented populations. Finally, the problems identified with the ‘Unconditional permission to eat’ subscale suggest that the items contained within this factor may not be as strongly related to the theoretical construct of intuitive eating as items within some of the other factors. All of these findings strongly suggest that use of the IES-2 in novel populations, particularly clinical settings, should ideally be preceded with a formal validation of the scale in the population of interest. Consideration should also be given as to whether the newer version of the scale (the IES-3) would better meet the needs of the intended populations.

### 5.4. Conclusion

In conclusion, the findings of this review demonstrate that the IES-2 has good convergent and known-groups construct validity but that there are several other important areas where the IES-2 underperforms psychometrically, raising doubts about the reliability and validity of the measure. Notably, there were problems with response distribution for some of the subscales, and correlations between the subscales and the total scale score were very low for ‘Unconditional permission to eat’ and ‘Body-food choice congruence’ (raising questions about how strongly they are related to intuitive eating as an overall concept). Importantly, the original four-factor structure of the IES-2 was also found to not be a good fit in many of the studies that assessed this and an alternative factor structure that excluded the ‘Unconditional permission to eat’ subscale was identified as a viable alternative. Other than studies focusing on weight or eating disorders, there was also only one clinical sample included in the review, which means there is insufficient evidence to infer how the measure would perform in medical populations. As a result of this and the other psychometric issues identified in this review, it is therefore recommended that researchers who wish to continue to use the IES-2 validate the scale in the population of interest before using it in novel clinical samples.

## Supporting information

S1 Table PRISMA 2020 checklist. (DOCX)

S2 Table Biopsychosocial constructs correlated with the IES-2. (DOCX)

S3 Table PRISMA 2020 for abstracts checklist. (DOCX)

S4 Table Extracted data table. (XLSX)

S1 File Database search terms. (DOCX)
