# Supplementary material for: A systematic review and narrative synthesis of the psychometric properties and biopsychosocial correlates of the English version of the Intuitive Eating Scale-2
Source: PLoS One. 2026 May 21;21(5):e0349590. doi: 10.1371/journal.pone.0349590 (PMC13193400; doi:10.1371/journal.pone.0349590)
Supplement: S2 Table — (DOCX) [file pone.0349590.s002.docx]

| Supporting Table 2 |  |  |
| --- | --- | --- |
| *Biopsychosocial constructs correlated with the IES-2* | | |
| Variable | Direction | Studies |
| Acceptance by others | Positive | Gan & Yeoh (2020); Modica & DeLillo (2022) |
| Aging, positive perception of | Positive | Frazier and Bezo Perez (2025) |
| Anxiety | Negative | Babbott et al. (2022); Barney et al. (2022); Dakin et al. (2024, vol. 201); Green and García (2025) |
| Appearance | Positive | Rodgers et al. (2022) |
| Acceptance of changes | Positive | Rodgers et al. (2022) |
| Evaluation | Positive | Tylka & Wood-Barcalow (2015); Tylka et al. (2022) |
| Masculine norms, conformity to | Positive | Sandler et al. (2023) |
| Muscular-ideal internalisation | Negative | Tylka et al. (2022) |
| Societal standards internalisation | Negative | Tylka & Wood-Barcalow (2015) |
| Thin-ideal internalisation | Negative | Tylka et al. (2022) |
| BMI | Negative | Anastasiades & Argyrides (2022); Braun, Unick et al. (2022); Craven & Fekete (2019); Henry et al. (2025); Jordan & Musher-Eizenman (2025); Linardon et al. (2020); Lovan et al. (2022); Modica & DeLillo (2022); Murray et al. (2023); Palascha et al. (2020); Smith et al. (2020); Soulliard & Vander Wal (2019); Swami et al. (2022); Teas et al. (2022); Tylka et al. (2015); Tylka et al. (2022); Webb & Hardin (2016) |
| Body |  |  |
| Acceptance | Positive | Modica & DeLillo (2022) |
| Acceptance by others | Positive | Gan & Yeoh (2020); Swami et al. (2022) |
| Aesthetic investment | Mixed | Murray et al. (2023) |
| Aesthetic satisfaction | Positive | Murray et al. (2023) |
| Appreciation | Positive | Anastasiades & Argyrides (2022); Brochu et al. (2024); Gan & Yeoh (2020); Jackson et al. (2024); Jordan & Musher-Eizenman (2025); Kelly & Stephen (2016); Linardon & Mitchell (2017); Linardon et al. (2022); Miller et al. (2018); Modica et al. (2023); Modica & DeLillo (2022); Murray et al. (2023); Palascha et al. (2020); Rodgers et al. (2022); Soulliard & Vander Wal (2019); Soulliard et al. (2025); Swami et al. (2022); Tylka & Wood-Barcalow (2015); Tylka et al. (2015); Tylka et al. (2022) |
| Awareness | Positive | Lovan et al. (2022) |
| Beliefs | Negative | Faw et al. (2021) |
| Checking | Negative | Linardon & Mitchell (2017) |
| Compassion | Positive | Sandler et al. (2023) |
| Concern | Negative | Keirns & Hawkins (2019, vol. 33) |
| Dissatisfaction | Negative | Frazier and Bezo Perez (2025); Liu et al. (2025); Tylka & Wood-Barcalow (2015) |
| Embodiment | Positive | Anastasiades & Argyrides (2022); Munroe et al. (2024) |
| Function (appreciation) | Positive | Anastasiades & Argyrides (2022); Brochu et al. (2024); Gan & Yeoh (2020); Jackson et al. (2024); Linardon et al. (2022); Modica & DeLillo (2022); Murray et al. (2023); Soulliard & Vander Wal (2019); Tylka et al. (2022) |
| Function investment | Mixed | Murray et al. (2023) |
| Function satisfaction | Positive | Murray et al. (2023) |
| Image acceptance | Positive | Soulliard & Vander Wal (2019) |
| Image flexibility | Positive | Linardon et al. (2022); Tylka et al. (2022); Webb & Hardin (2016) |
| Image, negative | Negative | Barney et al. (2022); Martin-Wagar & Heppner (2022) |
| Image, positive | Positive | Fitch et al. (2020); Kelly & Stephen (2016) |
| Image-related quality of life | Positive | Rodgers et al. (2022) |
| Intuition | Positive | Voelker et al. (2022) |
| Orientation, internal | Positive | Modica et al. (2023) |
| Responsiveness | Positive | Jeune et al. (2024) |
| Parts satisfaction | Positive | Sandler et al. (2023) |
| Satisfaction | Positive | Kelly & Stephen (2016); Miller et al. (2018); Rodgers et al. (2022) |
| Shame | Negative | Webb & Hardin (2016) |
| Shape concern | Negative | Nelson et al. (2023) |
| Shape preoccupation | Negative | Jackson et al. (2024) |
| Surveillance | Negative | Murray et al. (2023); Tylka & Wood-Barcalow (2015); Tylka et al. (2022) |
| Talk, negative | Negative | Jordan & Musher-Eizenman (2025) |
| Talk, positive | Positive | Jordan & Musher-Eizenman (2025) |
| Cognitive |  |  |
| Corumination | Negative | Faw et al. (2021) |
| Dichotomous thinking | Negative | Linardon & Mitchell (2017); Linardon et al. (2020) |
| Flexibility | Positive | Martin-Wagar & Heppner (2022) |
| Restraint |  | Lovan et al. (2022); Jeune et al. (2024); |
| Compensatory behaviour (purging) | Negative | Holmes et al. (2025) |
| Coping, proactive | Positive | Palascha et al. (2020); Tylka & Wood-Barcalow (2015) |
| Depression | Negative | Babbott et al. (2022); Barney et al. (2022); Green and García (2025); Martin-Wagar & Heppner (2022); Rodgers et al. (2022) |
| Eating |  |  |
| Anticipated enjoyment | Positive |  |
| Binge | Negative | Craven & Fekete (2019); Dakin et al. (2024, vol. 195); Holmes et al. (2025); Linardon & Mitchell (2017); Liu et al. (2025); Palascha et al. (2020); Tylka et al. (2015); Tylka et al. (2022) |
| Competence | Positive | Palascha et al. (2020); Rodgers et al. (2021) |
| Concern | Negative | Liu et al. (2025); Nelson et al. (2023) |
| Disinhibition | Negative | Dakin et al. (2024, vol. 195); Dakin et al. (2024, vol. 201); Linardon & Mitchell (2017) |
| Disordered | Negative | Babbott et al. (2022); Barney et al. (2022); Faw et al. (2021); Gan & Yeoh (2020); Gödde et al. (2022); Jordan & Musher-Eizenman (2025); Linardon et al. (2019); Martin-Wagar & Heppner (2022); Messer et al. (2021); Morris et al. (2024); Nelson et al. (2023); Smith et al. (2020); Tylka & Wood-Barcalow (2015) |
| Emotional, negative | Negative | Dakin et al. (2024, vol. 195); Jackson et al. (2024); Jeune et al. (2024); Lovan et al. (2022); Smith et al. (2020); Virani et al. (2020) |
| Emotional, positive | Mixed | Dakin et al. (2024, vol. 201) |
| Emotional overeating | Positive | Dakin et al. (2024, vol. 195); Dakin et al. (2024, vol. 201) |
| Emotional undereating | Positive | Dakin et al. (2024, vol. 195) |
| Externally motivated | Negative | Dakin et al. (2024, vol. 195); Jeune et al. (2024); Lovan et al. (2022) |
| For pleasure | Mixed | Dakin et al. (2024, vol. 201) |
| Flexible control | Negative | Linardon et al. (2019); Linardon et al. (2020); Tylka et al. (2015) |
| Fruit & vegetable intake | Mixed | Barad et al. (2019) |
| Guilt | Negative | Loor et al. (2023) |
| Inflexible control | Negative | Linardon et al. (2019) |
| Inflexible beliefs | Negative | Linardon et al. (2020) |
| Internally regulated | Positive | Palascha et al. (2020) |
| Restraint | Mixed | Dakin et al. (2024, vol. 195); Dakin et al. (2024, vol. 201); Gödde et al. (2022); Jackson et al. (2024); Kelly & Stephen (2016); Liu et al. (2025); Loor et al. (2023); Lovan et al. (2022); Miller et al. (2018); Nelson et al. (2023) |
| Restrictive | Negative | Palascha et al. (2020) |
| Rigid control | Negative | Linardon & Mitchell (2017); Linardon et al. (2019); Linardon et al. (2020); Tylka et al. (2015) |
| Self-regulation | Mixed | Dakin et al. (2024, vol. 195); Jeune et al. (2024) |
| Slowness | Positive | Palascha et al. (2020) |
| Uncontrolled | Negative | Jackson et al. (2024); Jeune et al. (2024); Lovan et al. (2022) |
| Emotion |  |  |
| Distress | Negative | Henry et al. (2025) |
| Dysregulation | Negative | Demi̇rgül & Ri̇gó (2023); Liu et al. (2025) |
| Mood | Positive | Loor et al. (2023) |
| Negative affect | Negative | Munroe et al. (2024); Tylka et al. (2015); Tylka et al. (2022) |
| Pessimism | Negative | Munroe et al. (2024) |
| Positive affect | Positive | Tylka et al. (2015) |
| Stress | Negative | Babbott et al. (2022); Green and García (2025) |
| Exercise |  |  |
| Driven | Negative | Liu et al. (2025) |
| Intrinsic motivation | Negative | Teas et al. (2022) |
| Motivation | Negative | Linardon & Mitchell (2017) |
| Variety | Negative | Voelker et al. (2022) |
| Food |  |  |
| Availability of healthy food | Positive | Ge et al. (2024) |
| Chips consumption | Negative | Tabatabai et al. (2021) |
| Diet quality | Positive | Jackson et al. (2024); Tabatabai et al. (2021) |
| Encouragement of diet diversity | Positive | Ge et al. (2024) |
| Fast food consumption | Negative | Tabatabai et al. (2021) |
| Fruit intake | Positive | Jackson, Sano et al. (2022) |
| Power of | Negative | Dakin et al. (2024, vol. 195) |
| Preoccupation | Negative | Tylka et al. (2015); Tylka et al. (2022) |
| Responsiveness | Negative | Dakin et al. (2024, vol. 195) |
| Restriction | Mixed | Tylka et al. (2022) |
| Sugar intake | Mixed | Jackson, Sano et al. (2022) |
| Vegetable intake | Mixed | Jackson, Sano et al. (2022) |
| Whole grains intake | Positive | Jackson, Sano et al. (2022) |
| Gender identity, non-affirmation of | Negative | Soulliard et al. (2025) |
| Gratitude | Positive | Tylka et al. (2022) |
| Hunger |  |  |
| Susceptibility to | Negative | Dakin et al. (2024, vol. 195); Dakin et al. (2024, vol. 201) |
| Infant feeding style | Mixed | Khalsa, Woo et al. (2019) |
| Interoceptive awareness | Positive | Jeune et al. (2024); Lovan et al. (2022) |
| Poor | Negative | Tylka et al. (2015) |
| Life satisfaction | Mixed | Munroe et al. (2024); Palascha et al. (2020); Tylka et al. (2015) |
| Medical readings |  |  |
| C-reactive proteins | Positive | Teas et al. (2022) |
| LDL/HDL ratio | Positive | Teas et al. (2022) |
| Triglycerides | Positive | Teas et al. (2022) |
| Medical symptoms/impairment | Negative | Linardon et al. (2019); Lovan et al. (2022) |
| Gastrointestinal | Mixed | Martin-Wagar & Heppner (2022) |
| Menopause | Negative | Frazier and Bezo Perez (2025) |
| Orthorexia |  | Demi̇rgül & Ri̇gó (2023) |
| Behaviours | Negative | Rodgers et al. (2021) |
| Healthy | Positive | Anastasiades & Argyrides (2022) |
| Nervosa | Negative | Anastasiades & Argyrides (2022) |
| Psychosocial impairment | Negative | Nelson et al. (2023) |
| PTSD symptoms | Negative | Holmes et al. (2025) |
| Relationship satisfaction | Positive | Faw et al. (2021) |
| Satiety |  |  |
| Honouring feelings of | Positive | Ge et al. (2024) |
| Responsiveness | Positive | Dakin et al. (2024, vol. 195); Palascha et al. (2020) |
| Self-compassion | Positive | Fitch et al. (2020); Kelly & Stephen (2016); Liu et al. (2025); Munroe et al. (2024); Webb & Hardin (2016) |
| Fear of | Negative | Sandler et al. (2023) |
| Self-esteem | Positive | Gan & Yeoh (2020); Gödde et al. (2022); Green and García (2025); Kelly & Stephen (2016); Palascha et al. (2020); Sandler et al. (2023); Swami et al. (2022); Tylka & Wood-Barcalow (2015) |
| Social support | Negative | Frazier and Bezo Perez (2025) |
| Weight | Negative | Fitch et al. (2020) |
| Bias, internalised | Negative | Brochu et al. (2024); Webb & Hardin (2016) |
| Change | Positive | Nelson et al. (2023); Palascha et al. (2020) |
| Concern | Negative | Gödde et al. (2022); Nelson et al. (2023) |
| Cycling severity | Negative | Palascha et al. (2020) |
| Contingent self-worth | Negative | Faw et al. (2021) |
| Overvaluation | Negative | Linardon & Mitchell (2017); Liu et al. (2025) |
| Shame and guilt | Negative | Craven & Fekete (2019) |
| Stigma, internalised | Negative | Braun, Unick et al. (2022) |
