# Supplementary material for: A systematic review and narrative synthesis of the psychometric properties and biopsychosocial correlates of the English version of the Intuitive Eating Scale-2
Source: PLoS One. 2026 May 21;21(5):e0349590. doi: 10.1371/journal.pone.0349590 (PMC13193400; doi:10.1371/journal.pone.0349590)
Supplement: S1 File — (DOCX) [file pone.0349590.s005.docx]

**Search terms**

**Scopus**

TITLE-ABS-KEY ( "intuitive eating" OR "intuitive eating scale-2" OR "intuitive eating scale 2" OR "IES-2" OR "IES2" ) AND PUBYEAR > 2012

**Web of Science**

"intuitive eating" OR "intuitive eating scale-2" OR "intuitive eating scale 2" OR "IES-2" OR "IES2" (Title) OR "intuitive eating" OR "intuitive eating scale-2" OR "intuitive eating scale 2" OR "IES-2" OR "IES2" (Abstract) OR "intuitive eating" OR "intuitive eating scale-2" OR "intuitive eating scale 2" OR "IES-2" OR "IES2" (Keyword Plus ®)

Timespan: 2013-01-01 to 2025-06-03 (Publication Date)

**PsycINFO**

TI ("intuitive eating" OR "intuitive eating scale-2" OR "intuitive eating scale 2" OR "IES-2" OR "IES2") OR AB ("intuitive eating" OR "intuitive eating scale-2" OR "intuitive eating scale 2" OR "IES-2" OR "IES2") OR KW ("intuitive eating" OR "intuitive eating scale-2" OR "intuitive eating scale 2" OR "IES-2" OR "IES2")

Timespan: 2013-01-01 to 2025-06-03 (Publication Date)

**MEDLINE**

(( "intuitive eating"[Title/Abstract] OR "intuitive eating scale-2"[Title/Abstract] OR "intuitive eating scale 2"[Title/Abstract] OR "IES-2"[Title/Abstract] OR "IES2"[Title/Abstract]) AND (("2013/01/01"[Date - Publication] : "3000"[Date - Publication]))
